# A Redox-Sensitive Luciferase Assay for Determining the Localization and Topology of Endoplasmic Reticulum Proteins

**DOI:** 10.1371/journal.pone.0035628

**Published:** 2012-04-18

**Authors:** Hai-Yin Li, Xue-Ming Zheng, Mei-Xia Che, Hong-Yu Hu

**Affiliations:** State Key Laboratory of Molecular Biology, Institute of Biochemistry and Cell Biology, Shanghai Institutes for Biological Sciences, Chinese Academy of Sciences, Shanghai, China; Ecole Polytechnique Federale de Lausanne, Switzerland

## Abstract

Correct localization and transmembrane topology are crucial for the proteins residing and functioning in the endoplasmic reticulum (ER). We have developed a rapid and convenient assay, based on the redox-sensitive luciferase from *Gaussia princeps* (Gluc) and green fluorescence protein (GFP), to determine the localization or topology of ER proteins. Using the tandem Gluc-GFP reporter fused to different positions of a target protein, we successfully characterized the topologies of two ER transmembrane proteins Herp and HRD1 that are involved in the ER quality control system. This assay method may also be applicable to the proteins in secretory pathway, plasma membrane, and other compartments of cells.

## Introduction

Eukaryotic endoplasmic reticulum (ER) serves many essential functions, including protein folding, disulfide bond formation, transport and secretion, glycosylation, and membrane integration [1], [2]. The ER resident proteins must adopt specific topology to achieve their proper functions. However, the techniques currently available for determining ER protein location and topology are limited. Protease protection assay is to engineer epitopes, such as Myc and FLAG, into a protein of interest, and then to assess its sensitivity to protease digestion in the absence or presence of detergents [3]. Another approach is glycosylation scanning, in which different glycosylation sites can be introduced into the full-length protein [4]. Recently, many approaches, based on fluorescent proteins and confocal microscopy, have become widely used in this purpose [5], [6], [7], [8]. Using redox-sensitive GFP (roGFP), ratiometric imaging was applied in plants to discriminate the orientation of roGFP relative to the ER membrane, as the roGFP fluorescence alters with different redox potentials across the ER membrane [6]. However, widespread knowledge of the location and topology of eukaryotic ER proteins demands rapid and convenient approaches.

*Gaussia* luciferase (Gluc), first isolated from the copepod marine organism *Gaussia princep*, is a monomeric enzyme consisting of 185 amino-acid residues (∼20 kDa) that does not require cofactors to be active [9]. Gluc catalyzes oxidation of the substrate coelenterazine in a reaction that produces light with a peak at 480 nm [9]. The substrate can permeate cell membranes and diffuse into all cellular compartments, allowing quantitative analysis in living cells. The human codon-optimized form of Gluc can be efficiently expressed in mammalian cells, generating a bioluminescent signal over 100-fold higher than those of *Renilla* and *Photinus* luciferases in cell lysates [9], [10]. Gluc is found to be naturally secreted, requiring an oxidative environment in the eukaryotic ER to form five putative disulfide bonds for its full activity [9], [10]. The bioluminescent signal of Gluc modified for retention in the ER is at least 10-fold higher than that confined in the cytoplasm [9], but the protein levels of Gluc fusions are variable due to expression and stability, which would limit the application of this reporter to the living cells.

Here, we report using a tandem Gluc-GFP (GG) as a redox-sensitive reporter to determine the orientation and topology of ER proteins. This newly developed assay method has been successfully applied to determine the orientation and topology of single-, double- and multiple-pass transmembrane proteins in ER.

## Results

### Disulfides and redox properties of Gluc

**Figure 1:**
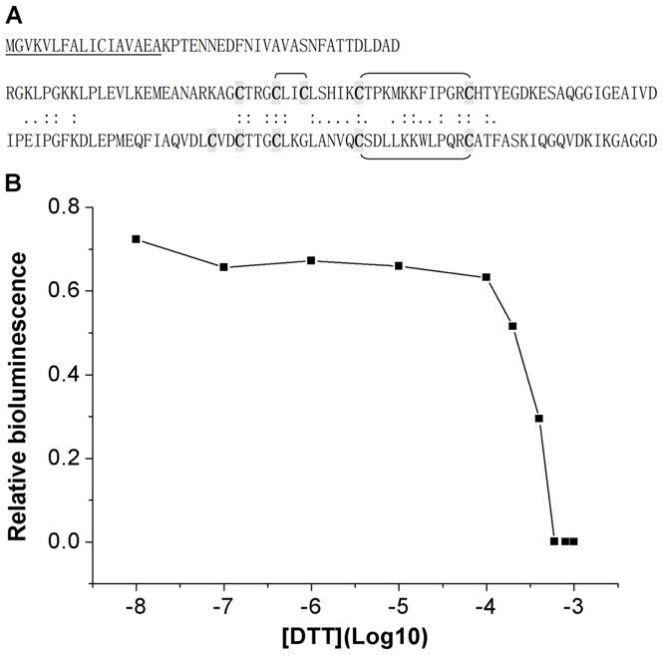
The bioluminescence activity of Gluc is sensitive to its redox environment. *A*, Amino-acid sequence of Gluc showing two homogenous subdomains and the disulfides. Three of the five disulfide bonds have been characterized by mass spectrometry (unpublished data). The 17-residue signal peptide is underlined. *B*, The bioluminescent activity of Gluc is DTT concentration dependent. The purified Gluc protein was incubated with different concentrations of DTT for 6 hrs at room temperature. The activity was represented as a percentage of the original Gluc activity without DTT. The bioluminescence decreases dramatically with the treatment of 0.1–1 mM DTT.

Gluc contains two homologous subdomains and ten cysteines that putatively form five disulfide bonds for its full activity (Fig. 1A) [11]. We overexpressed recombinant Gluc(-SP) (with deletion of the N-terminal signal peptide) enzyme in *E. coli* strain Origami B (DE3), which has a more oxidative cytoplasm. The purified Gluc(-SP) protein was incubated with different concentrations of DTT for measuring its sensitivity to the redox environment (Fig. 1B). The bioluminescence activity of Gluc(-SP) decreases dramatically with the treatment of 0.1–1 mM DTT, indicating that Gluc is a sensitive redox reporter. Thus the disulfide-bond dependent enzyme might be highly sensitive to the redox environment of ER, cytoplasm or other compartments of cells [12].

### Redox sensitivity of Gluc in cell compartments

To evaluate its redox sensitivity in human embryonic kidney 293T (HEK 293T) cells, the signal peptide of Gluc was deleted to confine Gluc(-SP) to the cytoplasm, while KDEL sequence was attached to the C-terminus of full-length Gluc for ER retention of Gluc-KDEL. For fusion expression with calnexin (CNX), a single-pass transmembrane ER protein [13], Gluc was attached to the lumenal N-terminus of CNX to generate Gluc-CNX, and the cytoplasmic C-terminus of CNX (CNX-Gluc). Gluc-CNX exhibits an activity approximately 6-fold higher than that of CNX-Gluc, while Gluc-KDEL does 10-fold higher than that of Gluc (-SP) (Fig. S1A). However, immunoblotting shows that these Gluc fusions exhibit similar ratios of protein amounts to those of bioluminescence (Fig. S1B). It would limit the application of this reporter to determine the localization or topology of ER proteins only by Gluc activity.

### Tandem Gluc-GFP as a reporter for determining the topologies of ER proteins

**Figure 2:**
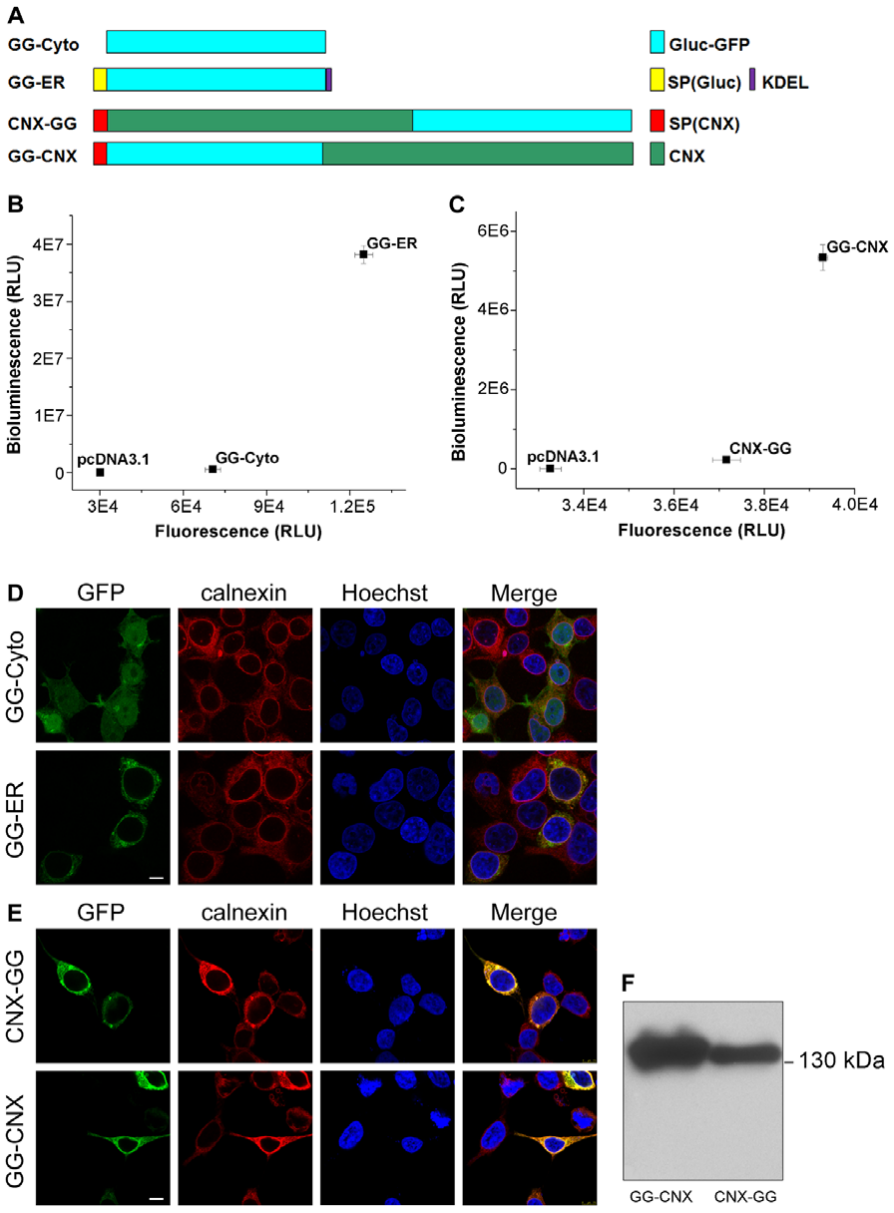
The Gluc-GFP reporter can be used to determine the localization and topology of ER proteins. *A*, Schematic representation of GG-Cyto, GG-ER, CNX-GG, and GG-CNX. SP(Gluc), signal peptide from Gluc; SP(CNX), signal peptide from CNX; KDEL, an ER-resident sequence. *B*, The bioluminescence/fluorescence plot of GG-ER and GG-Cyto. The relative bioluminescence activity of GG-ER is at least 10-fold higher than that of GG-Cyto. Data are represented as mean ± S.D. (n = 3). Results shown are representative of three repeat experiments. *C*, The bioluminescence/fluorescence plot of GG-CNX and CNX-GG. The relative bioluminescence activity of GG-CNX is at least 10-fold higher than that of CNX-GG. *D*, Confocal microscopy for localizations of GG-Cyto and GG-ER. Endogenous calnexin was used as ER membrane protein marker, while Hoechst was used to stain nuclei. Scale bar, 7.5 µm. *E*, As (D), confocal microscopy for localizations of GG-CNX and CNX-GG. *F*, Western blotting analysis for expression of GG-CNX and CNX-GG in HEK 293T cells. By quantifying the band intensities, the ratio of the protein amounts (GG-CNX/CNX-GG) is 1.8.

We have developed a reporter of tandem Gluc-GFP (GG) to determine the orientation of ER proteins. Involvement of green fluorescent protein (GFP) is for estimating the amounts of fusion proteins residing in each compartment. Firstly, we designed two constructs to test the feasibility of the redox reporter assay. In one construct, the signal peptide of Gluc was deleted to confine Gluc-GFP to the cytoplasm (GG-Cyto) (Fig. 2A). In another construct, with an N-terminal signal peptide and a C-terminal ER-resident KDEL sequence, Gluc-GFP was modified for retention in the ER lumen (GG-ER). These constructs were then transfected and the proteins were expressed in HEK 293T cells. As expected, under the same cell culture condition, the bioluminescence value of GG-ER is 1–2 orders of magnitude larger than that of GG-Cyto, while their fluorescence values are in the same order of magnitude (Fig. 2B). Fluorescence microscopy imaging indicates that they adopt correct subcellular localizations (Fig. 2D). Compared with endogenous calnexin, an ER membrane protein marker, GG-ER is really localized in the ER while GG-Cyto is in the cytoplasm, suggesting that the Gluc-GFP reporter has little influence on the correct localization of target proteins.

**Figure 3:**
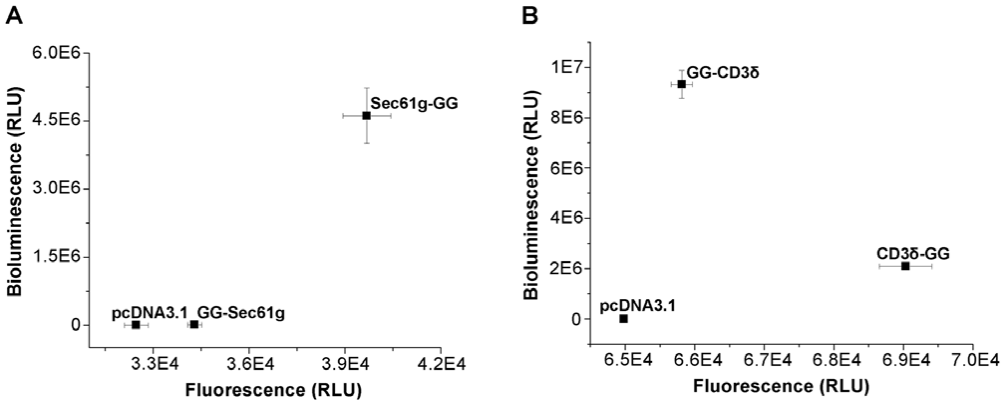
Tandem Gluc-GFP reporter for charactering the topology of single-pass transmembrane ER proteins. *A*, The bioluminescence/fluorescence plot of two Sec61g fusion forms. Sec61g-GG shows a bioluminescence at least 10-fold higher than that of GG-Sec61g. *B*, The bioluminescence/fluorescence plot of two CD3δ fusion forms. GG-CD3δ shows a bioluminescence at least 10-fold higher than that of CD3δ-GG. Data are presented as mean ± S.D. (n = 3). Results shown are representative of three repeat experiments.

Next, we applied the Gluc-GFP reporter to test the topology of calnexin, a single-pass transmembrane protein with its C-terminus facing the cytoplasm and its N-terminus residing in the ER [13]. We constructed two fusions of calnexin, one with Gluc-GFP attached to the lumenal N-terminus (GG-CNX), and the other with the reporter attached to the cytoplasmic C-terminus (CNX-GG). As in the model of GG-ER and GG-Cyto, the bioluminescence of GG-CNX is much higher than that of CNX-GG while their fluorescence values are within the same order (Fig. 2C), suggesting that the N-terminus of calnexin is localized in the ER lumen and the C-terminus is in the cytoplasm. Microscopic imaging exhibits that the exogenous Gluc-GFP fusions of calnexin have the same localizations as the endogenous calnexin (Fig. 2E), while immunoblotting analysis indicates their expression levels (Fig. 2F). These data demonstrate that the Gluc-GFP fused proteins are correctly expressed and localized in the cellular compartments. We also applied the Gluc-GFP reporter to examine the topology of Sec61g [14] and CD3δ [15]. The Gluc-GFP assay confirms that the N-terminus of Sec61g faces toward the cytoplasm while the C-terminus is in the ER (Fig. 3A). As for CD3δ, its N-terminus is localized in the ER while the C-terminus is in the cytoplasm (Fig. 3B). Collectively, these results from Gluc-GFP assay are well consistent with those from the previous studies by other techniques.

### Determining the topology of Herp, an ER transmembrane protein

**Figure 4:**
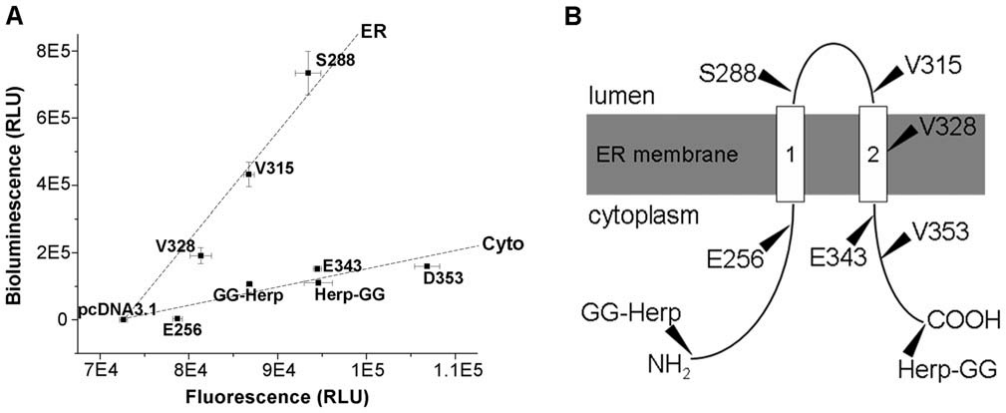
Determination of the transmembrane topology of Herp. *A*, The bioluminescence/fluorescence plot of the Gluc-GFP fusion forms of Herp. In the plot, the Gluc-GFP fusions localized in ER or cytoplasm are separately displaced in two groups as indicated with two dashed lines. Data are represented as mean ± S.D. (n = 3). Results shown are representative of three repeat experiments. *B*, Transmembrane topology of Herp. The solid lines denote the cytoplasmic or ER regions, while the rectangles represent the membrane-spanning regions. The residues pointed by triangles indicate the truncation positions for Gluc-GFP fusions.

To apply this Gluc-GFP assay to multi-pass transmembrane proteins involved in the ER quality control system, we investigated the topology of Herp, a membrane protein induced by ER stress [16]. It was previously proposed that Herp contains two transmembrane helices (Fig. S2A) [16], but the exact topology in ER membrane was unclear. We attached Gluc-GFP to the N-terminus (GG-Herp) and C-terminus (Herp-GG), and to several C-terminally truncated fragments to construct a series of fusion proteins. The assay shows that S288 and V315 fusions are localized in the ER lumen, while the N- and C-terminal fusions and three other fusions (E256, E343 and D353) are localized in the cytoplasm (Fig. 4A). The V328 fusion is an exception that it appears to be localized into the ER lumen, probably because V328 is buried in the ER membrane in its native state but resided in the ER in the fusion form due to inappropriate truncation of the transmembrane region. This result suggests that Herp spans ER membrane twice through the E256-S288 region and a proline-rich region V315-E343 near the C-terminal end (Fig. 4B). Note that, inconsistent with the prediction (Fig. S3A), the hydrophobic S288-V315 is not a transmembrane region but localized in the ER lumen.

### Determining the topology of HRD1, an ER transmembrane ubiquitin E3 ligase

**Figure 5:**
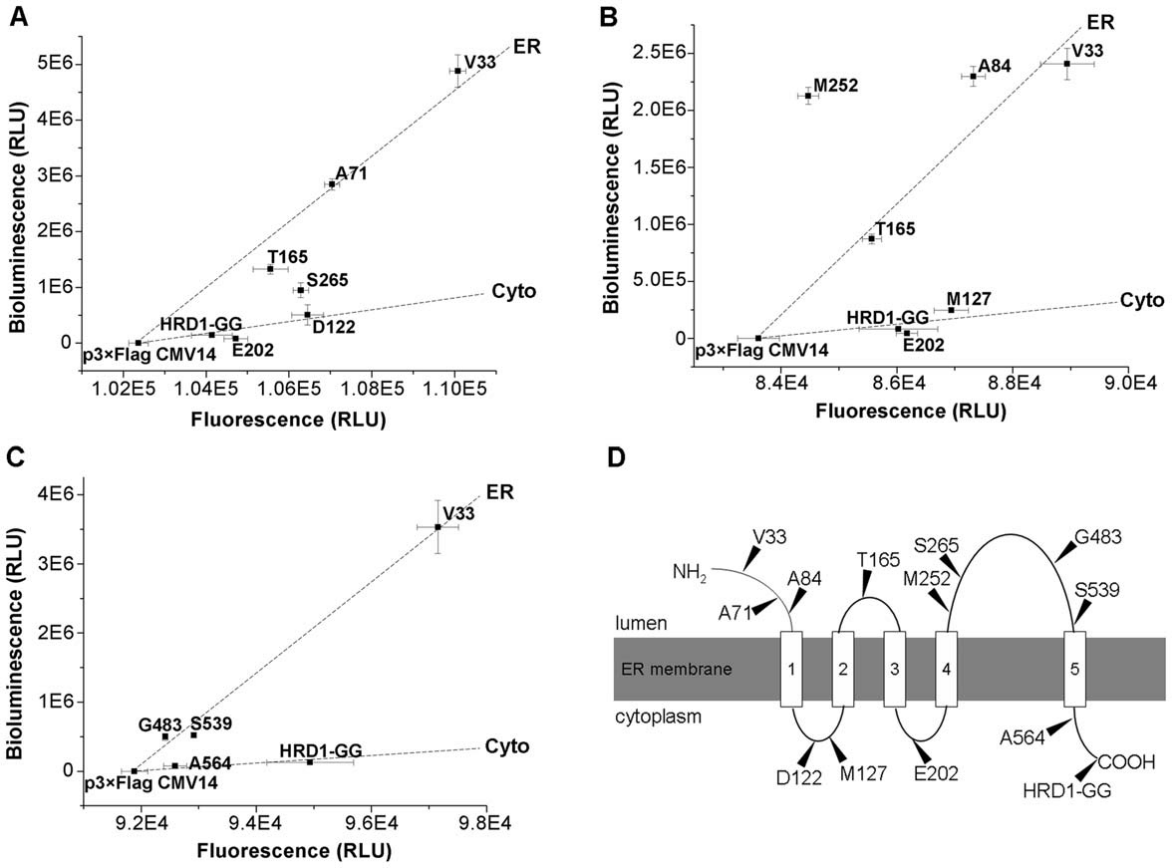
Determination of the transmembrane topology of HRD1. *A*, *B & C*, The bioluminescence/fluorescence plots of the Gluc-GFP fusion forms of HRD1. HRD1 and its C-terminally truncated fragments were fused with the Gluc-GFP reporter. In the plots, the dashed lines indicate that the Gluc-GFP fusions are localized in ER or cytoplasm. Data are represented as mean ± S.D. (n = 3). Results shown are representative of three repeat experiments. *D*, Transmembrane topology of HRD1. The solid lines denote the cytoplasmic or ER regions, while the rectangles represent the membrane-spanning regions. The residues pointed by triangles indicate the truncation positions for Gluc-GFP fusions.

HRD1 was proposed to be an essential component of ubiquitin ligase complex involved in the ER-associated degradation (ERAD) [17]. The hydropathy profile of HRD1 predicts the existence of six transmembrane domains [18] (Fig. S2B). We applied this newly developed assay to examine the multi-spanning membrane topology of HRD1 by analyzing both the bioluminescence and fluorescence of the Gluc-GFP fusions. A series of constructs include C-terminal fusion (HRD1-GG) and six C-terminal truncation fusions (Fig. 5A). The bioluminescence/fluorescence plot suggests that V33, A71 and T165 fusions are localized in the ER lumen, while the C-terminal, D122 and E202 fusions are resided in the cytoplasm. The obscure localization of S265 fusion might be caused by the improper truncation of the transmembrane domain. So we constructed additional fusions to verify the more refined orientations (Fig. 5B). The A84 fusion is localized in the ER lumen as V33 fusion, while M127 fusion is resided in the cytoplasm as the C-terminal fusion. Taken together, these results suggest that the N-terminal portion of HRD1 contains only four transmembrane regions located in A84-D122, M127-T165, T165-E202, and E202-M252 regions (Fig. 5D). Intriguingly, different from C-terminal HRD1-GG, the M252 fusion is localized in the ER lumen, giving rise to relatively higher bioluminescent signal than V33 fusion, suggesting there is another unexpected transmembrane region between M252 and the C-terminus (Fig. 5B). The hydropathy profile of HRD1 implies there is a hydrophobic region occurred near the C-terminus (Fig. S2B). We constructed three additional C-terminally truncated fragments fused with Gluc-GFP (G483, S539 and A564 fusions) (Fig. 5C). The data indicate that G483 and S539 fusions are localized in the ER lumen similar to V33 fusion, whereas A564 fusion is in the cytoplasm with the same side as the C-terminal fusion. Thus, compared with the previous model (Fig. S3B) [18], HRD1 contains an unpredicted transmembrane region of S539-A564 that confers the C-terminus resided in the cytoplasm (Fig. 5C & 5D).

## Discussion

Taking GG-ER and GG-Cyto as examples, the Gluc-GFP reporter has been successfully applied to investigate the localization of ER proteins. This research with the Gluc-GFP fusions also confirms the known topology of single-pass transmembrane ER proteins with different sizes and orientations, such as calnexin (Fig. 2C), Sec61g (Fig. 3A) and CD3δ (Fig. 3B). Although our data indicate that Herp spans the ER membrane twice as previously proposed [16], this research suggests that the second transmembrane domain is located in a proline-rich region but not in the hydrophobic region predicted by various algorithms (Fig. 4). The bioluminescence/fluorescence plots of HRD1 fusions suggest that HRD1 contains five transmembrane helices (Fig. 5). However, different from the previous model [18], there is an unpredicted transmembrane domain near the C-terminus that confers the C-terminus resided in the cytoplasm.

Our immunoblotting analysis has revealed that the amount of GG-CNX is about two-fold higher than that of CNX-GG (Fig. 2F), but the fluorescence intensity of GG-CNX is only 1.5-fold larger than that of CNX-GG (Fig. 2C). This suggests that the fluorescence brightness of GFP localized in ER lumen is a little lower than it in cytoplasm, as reported in the literature that the fluorescence of GFP slightly decreases in the relatively oxidative environment of ER lumen [19]. These phenomena that Gluc is highly active while GFP is less fluorescent in ER further facilitates the discrimination of lumenal and cytoplasmic orientation of ER proteins. Actually, the modified GFP for eukaryotic ER retention is commercially available and widely used [20].

Redox-sensitive GFP (roGFP) has been used to discriminate the lumenal and cytoplasmic orientation in plants [6]. Two cysteines were introduced into the appropriate positions of GFP to form a disulfide bond [21], so that the oxidized form of roGFP gives an excitation peak at 405 nm and the reduced at 488 nm. Due to the low excitation efficiency of the reduced form at 405 nm, in addition to ratiometric fluorescence imaging, cell-wall staining, signal-to-noise improvement and mathematical calculation were needed for this purpose [6]. New GFP-based redox sensors (roGFP1-iX) with midpoint potentials suited for relatively oxidizing subcellular compartments were also reported [21]; its derivative (RoGFP1-iL) has been applied to monitor the redox state in the mammalian endoplasmic reticulum [22]. The present study shows that Gluc is naturally matured in ER lumen for formation of the five disulfide bonds, which enables its high sensitivity to the variation of redox environments.

Correct localization and transmembrane topology are essential for ER proteins. The redox-sensitive luciferase assay described here provides a new approach with many advantages for determining protein localization and topology. This method does not require cell extraction or biochemical treatment. The use of Gluc-GFP reporter eliminates the need for appropriate and available antibodies or expensive microscope devices. These advantages make the approach become a promising application to high-throughput screening. Although Gluc is the smallest bioluminescent protein, in rare cases, the fusion of Gluc-GFP reporter may interfere with proper localization or topology of the target protein. In that case, more elaborate techniques that allow the separation of different protein sizes in gels after proteolysis may still be preferable. For many experimental systems, however, the straightforwardness and reliability of the Gluc-GFP reporter make it a valuable assay for determining the localization and transmembrane topology of ER proteins.

Based on the redox sensitivity, the Gluc-GFP reporter would be applicable to monitoring the oxidative microenvironment in Golgi apparatus, mitochondrion, dynamic protein transportation, secretory pathway and ER stress [10], and also to discriminating intracellular or extracellular orientation of plasma membrane proteins.

## Materials and Methods

### Plasmid constructions

The human codon-optimized DNA encoding Gluc protein (185 a.a.) was chemically synthesized (Fig. S4). The DNA of Gluc(-SP) (18–185) was inserted into prokaryotic expression vector pET-22b(+) (Novagen) using Nde I/Xho I sites for expression in *E. coli*. To generate a tandem reporter construct, GFP was attached directly to the C-terminus of Gluc (in which the N-terminal secretory signal peptide of 17 amino-acid residues was deleted). This tandem Gluc-GFP reporter is localized in the cytoplasm (GG-Cyto) due to lack of the signal peptide. For ER retention (GG-ER), the signal peptide of Gluc remained in the N-terminus, a Myc tag and a KDEL sequences were fused to the C-terminus. These fusions were inserted into the the BamH I/Xho I sites of mammalian expression vector pcDNA3.1/Myc-His (Invitrogen). For GG-CNX, the Gluc-GFP reporter was inserted between codon 25 and 26 of calnexin using EcoR I/EcoR V sites, but not to interfere with the N-terminal signal peptide of calnexin. Similarly, the Gluc-GFP reporter was attached to the C-terminus of calnexin using EcoR I site to construct CNX-GG. The calnexin fusions were also inserted into the BamH I/Xho I sites of pcDNA3.1/Myc-His. Using EcoRI site, Gluc-GFP was also attached to the N-terminus (GG-Herp), C-terminus (Herp-GG) of Herp, and to several C-terminally truncated fragments (E256, S288, V315, V328, E343, and D353), and these fusions were also inserted into the BamH I/Xho I sites of pcDNA3.1/Myc-His. The constructs for HRD1 included C-terminal fusion (HRD1-GG) and twelve C-terminally truncated fragment fusions (V33, A71, A84, D122, M127, T165, E202, M252, S265, G483, S539, and A564), attached with GG using EcoR I site. These fusions were inserted into the Hind III/BamH I-digested vector p3×FLAG-CMV-14 (Sigma).

### In vitro assay for the redox sensitivity of Gluc

The Gluc protein was overexpressed in *E. coli* strain Origami B (DE3) (Novagen) and purified on Ni^2+^-NTA agarose followed by Superdex 75 column chromatography (GE Healthcare). The purified Gluc protein was diluted to 10 µM in a buffer of 50 mM Tris (pH7.4) containing 100 mM NaCl and different concentrations of DTT, and incubated for 6 hrs at room temperature. The samples were diluted to a final concentration of 10 nM in 200 µL of PBS buffer (50 mM phosphate, 500 mM NaCl, pH7.8). Ten seconds after addition of coelenterazine to a final concentration of 1 µM, the bioluminescence measurements were performed on a GloMax 20/20 luminometer (Promega) with an integration time of 1 sec.

### Cell culture and transfection

Human embryonic kidney 293T (HEK 293T) cells (American Type Culture Collection, Manassas, VA, USA) were seeded into 24-well plate (Nunc) and cultured in Dulbecco's modified Eagle's medium (DMEM) containing 10% fetal bovine serum, 100 units/mL penicillin and 100 µg/mL streptomycin at 37°C under humidified air containing 5% CO_2_. We performed transient transfection of cells using the FuGene HD transfection reagent (Roche) according to the manufacturer's instructions. Cells were subjected to analysis 36 hrs after transfection. For microscopic imaging, cells were plated on poly-L-lysine-coated coverslips.

### Fluorescence and bioluminescence measurements

Cells were detached and suspended in 100 µL PBS (50 mM phosphate, pH7.8) containing 500 mM NaCl and distributed into a black 96-well plate (Greiner Microlon). Fluorescence and bioluminescence measurements were performed with a Mithras LB 940 multimode reader (Berthold Technologies). We measured the fluorescence using an excitation filter at 485 nm and an emission filter at 510 nm (lamp energy 50000, counting time 1 sec) and determined luminescence 5 sec after addition of coelenterazine (20 µL, 1 µM) (Promega) using an automatic injector.

### Microscopy imaging and immunoblotting analysis

Cells were fixed with 4% paraformaldehyde for 10 min, permeabilized with 0.1% Triton X-100, and blocked with 1% bovine serum albumin and 10% fetal bovine serum. The cells were then immunostained with a primary antibody against calnexin, followed by a TRITC-conjugated secondary antibody and Hoechst (a DNA-specific fluorescent dye). Confocal laser scanning microscopic analysis was performed on a Leica TCS SP2 confocal laser imaging system with a 63×/1.4 oil immersion objective (Leica Microsystems). Cells expressing GG-CNX, CNX-GG or other fusions were lysed and subjected to standard immunoblotting using an anti-GFP antibody.

## Supporting Information

Figure S1 **The bioluminescence of differently localized Gluc enzyme.** (A) The bioluminescence activities of Gluc in different locations. Gluc-CNX exhibits an activity approximately 6-fold higher than that of CNX-Gluc, while Gluc-KDEL does 10-fold higher than that of Gluc(-SP). Gluc(-SP), Gluc protein without the signal peptide. (B) Immunoblotting showing expression of the Gluc forms. Lane 1, Gluc-CNX; lane 2, CNX-Gluc; lane 3, Gluc-KDEL; and lane 4, Gluc(-SP). All forms of Gluc were correctly expressed in HEK 293T cells.
(DOC)

Figure S2 **Kyte-Doolittle hydropathy plots of Herp (A) and HRD1 (B).** The window for the prediction spans 19 amino-acid residues. The putative transmembrane regions are numbered above the plots.
(DOC)

Figure S3 **Prediction of the topologies of Herp (A) and HRD1 (B) by TOPCONS.** Predicted topologies by different methods and TOPCONS consensus prediction are shown. Z-coord, predicted distance to the membrane center (Z = 0); ΔG value, predicted free energy of insertion of a transmembrane (TM) helix into the membrane of the endoplasmic reticulum. (A) Predicted TM helices of Herp: 265–285, 287–307, 362–382. (B) Predicted TM helices of HRD1: 4–24, 41–61, 99–119, 136–156, 170–190, 225–245.
(DOC)

Figure S4 **The DNA and protein sequences of Gluc (185 a.a.).** The human codon-optimized DNA sequence encoding the full-length Gluc protein was chemically synthesized by our laboratory.
(DOC)
