# Supplementary material for: A Redox-Sensitive Luciferase Assay for Determining the Localization and Topology of Endoplasmic Reticulum Proteins
Source: PLoS One. 2012 Apr 18;7(4):e35628. doi: 10.1371/journal.pone.0035628 (PMC3329452; doi:10.1371/journal.pone.0035628)
Supplement: Figure S1 — The bioluminescence of differently localized Gluc enzyme. (A) The bioluminescence activities of Gluc in different locations. Gluc-CNX exhibits an activity approximately 6-fold higher than that of CNX-Gluc, while Gluc-KDEL does 10-fold higher than that of Gluc(-SP). Gluc(-SP), Gluc protein without the signal peptide. (B) Immunoblotting showing expression of the Gluc forms. Lane 1, Gluc-CNX; lane 2, CNX-Gluc; lane 3, Gluc-KDEL; and lane 4, Gluc(-SP). All forms of Gluc were correctly expressed in HEK 293T cells. (DOC) [file pone.0035628.s001.doc]

**Figure S1**


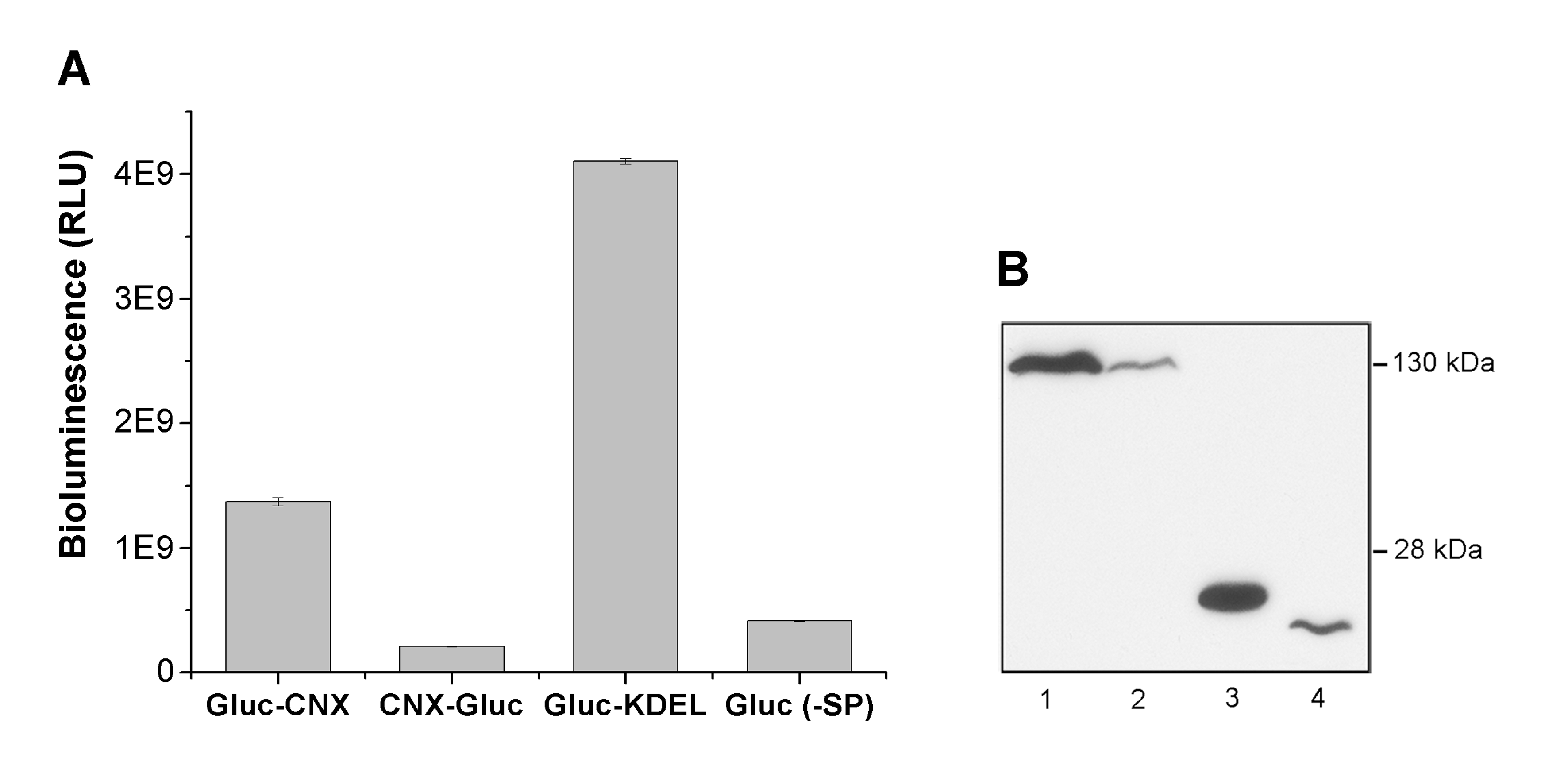


***Supplemental Figure S1.*** The bioluminescence of differently localized Gluc enzyme. (A) The bioluminescence activities of Gluc in different locations. Gluc-CNX exhibits an activity approximately 6-fold higher than that of CNX-Gluc, while Gluc-KDEL does 10-fold higher than that of Gluc(-SP). Gluc(-SP), Gluc protein without the signal peptide. (B) Immunoblotting showing expression of the Gluc forms. Lane 1, Gluc-CNX; lane 2, CNX-Gluc; lane 3, Gluc-KDEL; and lane 4, Gluc(-SP). All forms of Gluc were correctly expressed in HEK 293T cells.
