# Supplementary material for: A Redox-Sensitive Luciferase Assay for Determining the Localization and Topology of Endoplasmic Reticulum Proteins
Source: PLoS One. 2012 Apr 18;7(4):e35628. doi: 10.1371/journal.pone.0035628 (PMC3329452; doi:10.1371/journal.pone.0035628)
Supplement: Figure S2 — Kyte-Doolittle hydropathy plots of Herp (A) and HRD1 (B). The window for the prediction spans 19 amino-acid residues. The putative transmembrane regions are numbered above the plots. (DOC) [file pone.0035628.s002.doc]

**Figure S2**

**
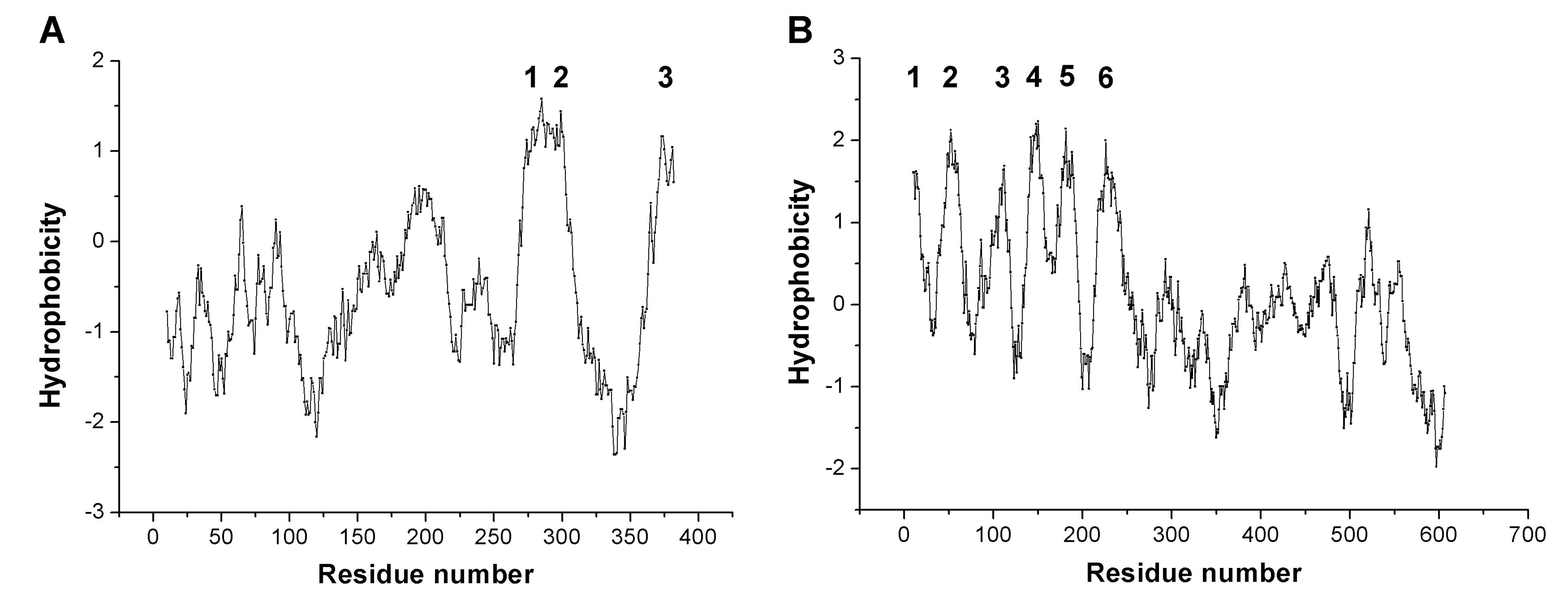
**

***Supplemental Figure S2.*** Kyte-Doolittle hydropathy plots of Herp (A) and HRD1 (B). The window for the prediction spans 19 amino-acid residues. The putative transmembrane regions are numbered above the plots.

**Reference**

Kyte J, Doolittle RF (1982) A simple method for displaying the hydropathic character of a protein. J Mol Biol 157: 105-132.
