# Supplementary material for: A Redox-Sensitive Luciferase Assay for Determining the Localization and Topology of Endoplasmic Reticulum Proteins
Source: PLoS One. 2012 Apr 18;7(4):e35628. doi: 10.1371/journal.pone.0035628 (PMC3329452; doi:10.1371/journal.pone.0035628)
Supplement: Figure S3 — Prediction of the topologies of Herp (A) and HRD1 (B) by TOPCONS. Predicted topologies by different methods and TOPCONS consensus prediction are shown. Z-coord, predicted distance to the membrane center (Z = 0); ΔG value, predicted free energy of insertion of a transmembrane (TM) helix into the membrane of the endoplasmic reticulum. (A) Predicted TM helices of Herp: 265–285, 287–307, 362–382. (B) Predicted TM helices of HRD1: 4–24, 41–61, 99–119, 136–156, 170–190, 225–245. (DOC) [file pone.0035628.s003.doc]

**Figure S3**


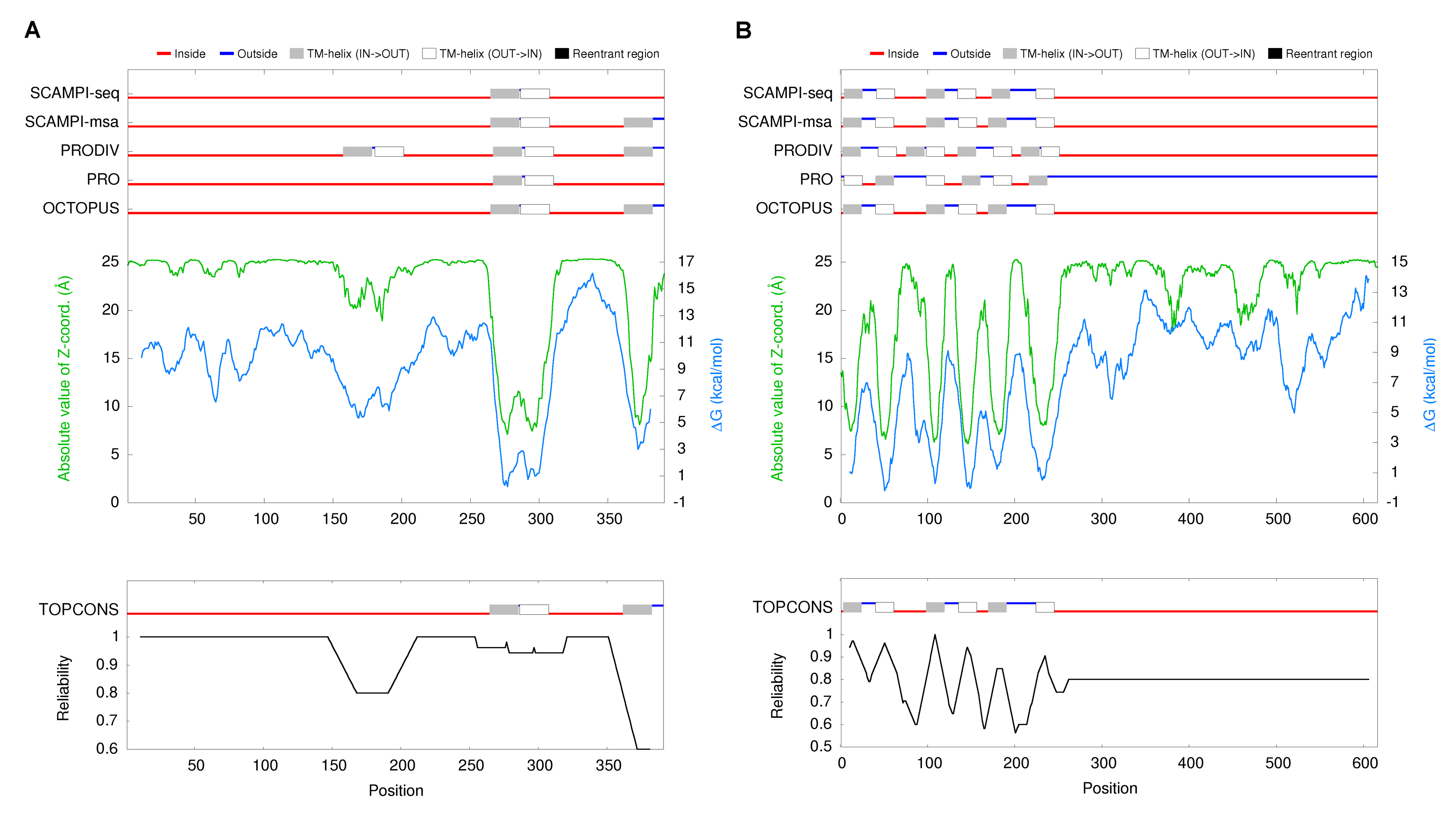


***Supplemental Figure S3*** Predicting the topologies of Herp (A) and HRD1 (B) by TOPCONS. The predicted topologies by different methods and TOPCONS consensus prediction are shown. Z-coord, predicted distance to the membrane center (Z=0); ΔG value, predicted free energy of insertion of a transmembrane (TM) helix into the membrane of the endoplasmic reticulum. (A) Predicted TM [helices](http://www.iciba.com/helixes/) of Herp: 265-285, 287-307, 362-382. (B) Predicted TM [helices](http://www.iciba.com/helixes/) of HRD1: 4-24, 41-61, 99-119, 136-156, 170-190, 225-245.

**References**

1. Bernsel A, Viklund H, Hennerdal A, Elofsson A (2009) TOPCONS: consensus prediction of membrane protein topology. Nucleic Acids Res 37: W465-468.

2. Bernsel A, Viklund H, Falk J, Lindahl E, von Heijne G, et al. (2008) Prediction of membrane-protein topology from first principles. Proc Natl Acad Sci U S A 105: 7177-7181.

3. Viklund H, Elofsson A (2008) OCTOPUS: improving topology prediction by two-track ANN-based preference scores and an extended topological grammar. Bioinformatics 24: 1662-1668.

4. Hessa T, Meindl-Beinker NM, Bernsel A, Kim H, Sato Y, et al. (2007) Molecular code for transmembrane-helix recognition by the Sec61 translocon. Nature 450: 1026-1030.

5. Granseth E, Viklund H, Elofsson A (2006) ZPRED: predicting the distance to the membrane center for residues in alpha-helical membrane proteins. Bioinformatics 22: e191-196.

6. Viklund H, Elofsson A (2004) Best alpha-helical transmembrane protein topology predictions are achieved using hidden Markov models and evolutionary information. Protein Sci 13: 1908-1917.
