# Supplementary material for: A Redox-Sensitive Luciferase Assay for Determining the Localization and Topology of Endoplasmic Reticulum Proteins
Source: PLoS One. 2012 Apr 18;7(4):e35628. doi: 10.1371/journal.pone.0035628 (PMC3329452; doi:10.1371/journal.pone.0035628)
Supplement: Figure S4 — The DNA and protein sequences of Gluc (185 a.a.). The human codon-optimized DNA sequence encoding the full-length Gluc protein was chemically synthesized by our laboratory. (DOC) [file pone.0035628.s004.doc]

**Figure S4**


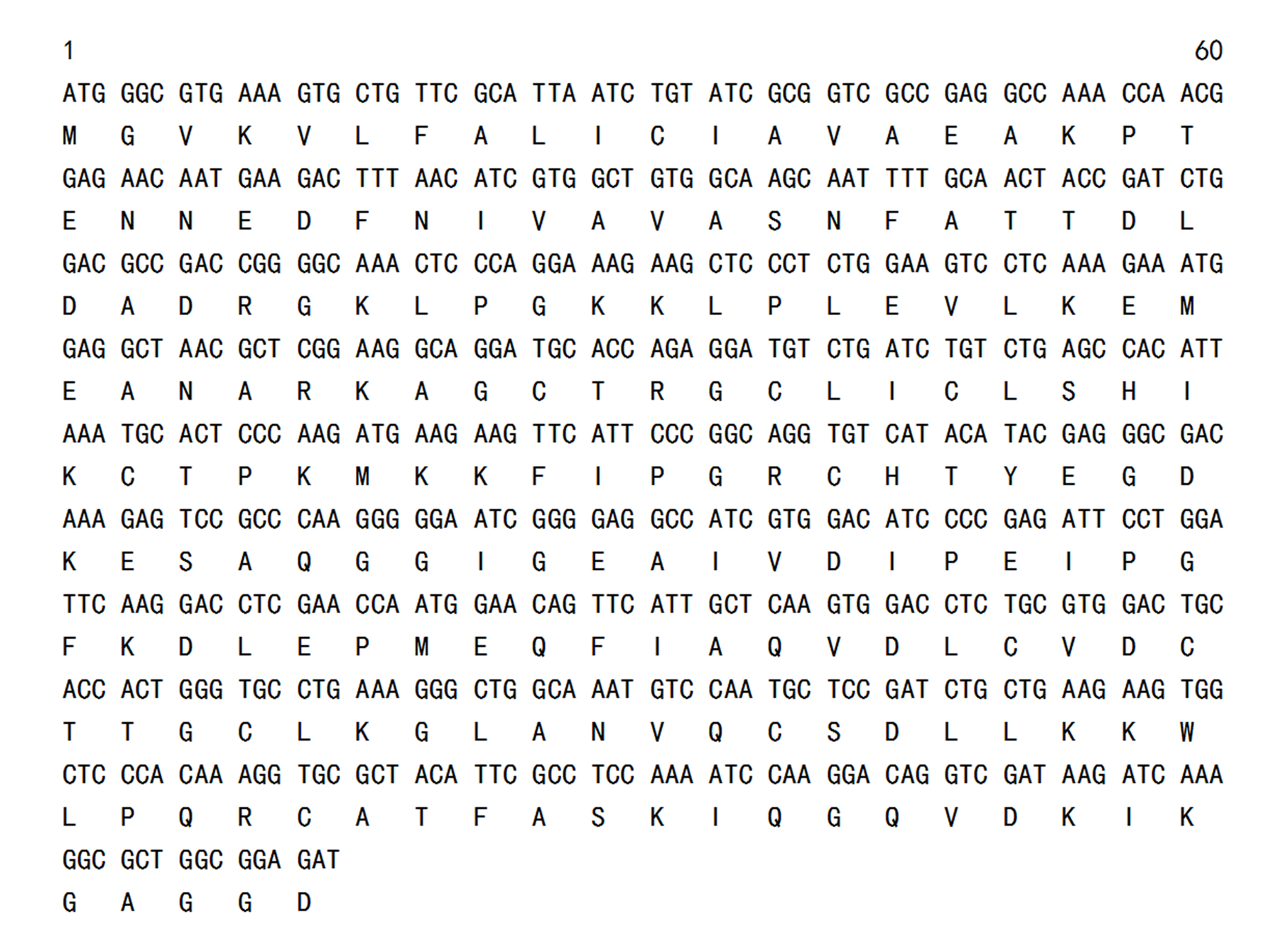


***Supplemental Figure S4*** The DNA and protein sequences of Gluc (185 a.a.). The human codon-optimized DNA sequence encoding the full-length Gluc protein was chemically synthesized by our laboratory.
